# Collateral damage: has the COVID-19 pandemic more strongly impacted medical research than other scientific areas?

**DOI:** 10.7717/peerj.15436

**Published:** 2023-06-13

**Authors:** Alejandro Farji-Brener, Sabrina Amador-Vargas

**Affiliations:** 1 CRUB, Universidad Nacional del Comahue, Bariloche, Argentina; 2 CONICET, LIHO, Inibioma, Bariloche, Argentina; 3 Smithsonian Tropical Research Institute, Panama, Panama

**Keywords:** COVID-19, Resource allocation, Scientific production, Funding

## Abstract

The principle of resource allocation states that diversion of resources to attend a function may compromise others. The COVID-19 pandemic required a rapid response with a justifiable relocation of equipment, funds and human resources. Based on the ecological principle of allocation, we tested whether the relocation of resources to support COVID-19 research was more detrimental to medical research than to research in other scientific areas. We compared the yearly number of published articles from 2015 to 2021 using disease-related keywords and non-medical scientific keywords. Contrary to the expectation, we found an abrupt reduction in the publication rates in all research areas from 2019 to 2020 or 2021, compared to the pre-pandemic period (2015–2019). The allocation effect on medical research may be overshadowed by stronger effects of the pandemic, or it may become evident in the coming years. The drastic reduction in published papers could have negative consequences for scientific advancements, including understanding and curing diseases other than COVID-19 that strongly affect humanity.

## Introduction

The principle of allocation, the assignation of limited resources in certain activities at the expense of others, is a basic concept in ecology that applies to other disciplines (Lloyd, 1988). For example, in plants the allocation of resources to defenses compromises growth (Herms & Mattson, 1992), and animals that invests in survival or growth can end up having fewer resources for reproduction (Heino & Kaitala, 1999). These tradeoffs apply to other disciplines, such as conservation planning and the organization of companies, where funds must be distributed among different activities (Elster, 1992; Joseph, Maloney & Possingham, 2009). Consequently, the most successful organisms or organizations typically are those that best balance conflicting demands. Nations are not the exception. As resources are often scarce, governments must make challenging decisions about how to allocate the limited resources to satisfy the several demands of societies.

A pandemic aggravates the governments’ dilemma of limited resource allocation. In a normal context, governments often diversify the investment among different scientific research programs, and scientists perform research in a wide range of topics. However, a pandemic forces governments and institutions to reassign funds, and to shift the priority to the most urgent activities. The pandemic of the 2019 coronavirus disease (COVID-19) has led to a substantial increase in the demands for research on this virus worldwide. Governments and funding agencies across the world reassigned financial and technical support for research initiatives related to SARS-CoV-2 (Andrijevic et al., 2020), and several public and private health research institutes reorganized their facilities, structure and operations to work collaboratively toward improving the diagnosis and understanding of this virus. However, this positive and necessary redistribution of funding and human resources may affect the research programs of other important human diseases, as the allocation principle predicts. COVID-19 is not the only disease afflicting millions of humans around the world. For example, more than 400 million people suffer diabetes worldwide today, and the closely correlated cardiovascular diseases remain the main cause of death in Western societies (Zeggini et al., 2020). Human immunodeficiency virus (HIV) and AIDS, tuberculosis (TB) and malaria together accounted for about 5% of all causes of death worldwide (Prudêncio & Costa, 2020). Only in 2019, stroke, cancer, Alzheimer and diarrhea together accounted for approximately 12 million deaths, according the World Health Organization. In sum, humans continue to be afflicted by numerous other devastating diseases besides COVID-19.

Several lines of evidence suggest that COVID-19 research may have negatively affected research programs of other key diseases. The reasons include a sharp redirection of resources towards COVID-19 at the expense of non-COVID-19 research, the consequences of lockdown and the closure of research centers and institutions, the suspension of non-COVID clinical trials, supply shortages, and academic researchers now full time in clinical duties, among others (Chan & Tan, 2020; Kardas-Nelson, 2020; Palacios Huatuco et al., 2021; Yanow & Good, 2020). For example, the leading UK charity Cancer Research UK cut the funding for research by USD 54 million, in response to huge fundraising shortfalls caused by the COVID-19 pandemic (Iacobucci, 2020). UK Research and Innovation, the national funding agency for science and research in the UK, has moved to refocus its activities and to streamline applications for new research, including publishing guidance for researchers who currently hold grants, on how they might repurpose their funds for COVID-19 (Iacobucci, 2020). The American pharmaceutical companies’ trade group PhRMA says that more than half of its members have committed research and development funds toward COVID-19 treatments and vaccines. That means cutting funding from other existing projects (Yang, 2020). All the evidence suggests that the research, publications and new trials in diseases other than COVID-19, or its study independently from COVID-19 will most probably decrease on short and long-term due to this pandemic (Omary & Hassan, 2020; Zeggini et al., 2020). These arguments seem logical, but we lack empirical data.

Here we tested whether the redistribution of funds, equipment and human resources toward studying COVID-19 negatively affected the research in other diseases, as the allocation principle predicts. Therefore, we compared the publication rate on COVID-19 to that of other diseases in pre- and pandemic periods. We also analyzed the scientific production in other scientific disciplines as a control, aiming to understand the overall effect of the pandemic on scientific production. If the reorientation of resources toward COVID-19 negatively impacted mostly other health research programs, we expected that a vast increment in scientific literature about SARS-CoV-2 during the pandemic period would simultaneously occur with a strong decrease in the rate of scientific publications regarding other human diseases; for other scientific disciplines, we expected a smaller effect of the pandemic on the publication rate, assuming funding for each area is independent, or at least the first funds to suffer from allocation principle would be those in the medical area, before directly compromising other research areas.

## Materials and Methods

We searched in the Web of Science database (all collections) for the keyword “coronavirus”, 14 medicine-related keywords and 14 control keywords (non-medicine related; Table S1), filtered by years (from 2015 to 2021). Medicine-related keywords include words among the top 10 causes of death for the year 2019 (WHO, 2020), neglected tropical diseases (CDC, 2022), and other important diseases not listed in the previous two sources (*e.g*., cancer). Control keywords were chosen from diverse scientific areas, exploring topics from textbooks and encyclopedias to find words not immediately associated with medicine or human health. These words represent broad topics in other disciplines or more specific subjects, and are likely to be mentioned in publications not related to medicine. Those words could be mentioned in the text of medicine papers, but it is unlikely they will be retrieved by our search (Web of Science results searches among Topic, Title, Abstract and indexing). Search terms with multiple words were written in quotes, *e.g*., “diabetes mellitus”, so it searches for both words together and not each word separately. We also filtered by document type, choosing “Articles” only. We searched the Web of Science because it is a trusted database, it covers a broader range of topics than other databases (*e.g*., PubMed), it is more accurate to pinpoint research articles than Google Scholar, and it is weekly updated (Falagas et al., 2008).

We then searched again for the year 2020 and 2021, using the same list of keywords but adding “AND covid” (*e.g*., “pulmonary disease” AND covid), to get the number of records that were related to the subject and COVID. To obtain the number of articles published in 2020 and 2021 unrelated to COVID, we subtracted the number of papers obtained in the last search (keyword AND covid, about 3.5 ± 5% of the papers) from the total articles for that keyword in 2020 and 2021. This approach may cause a slight underestimation of the number of in a particular topic, because some may have been excluded because they used the word COVID in the abstract, title or keywords, even when COVID was not part of the research. Then, we calculated the rate of change from one year to the next, as the subtraction of a year’s articles minus the previous year’s articles divided by the latter number. For instance, the 2015–2016 rate of change would be the articles of 2016 minus the articles of 2015, divided by the articles of 2015. Therefore, we had four pre-pandemic rates of change in research article production (2015–16, 2016–17, 2017–18, 2018–19) to compare to the two pandemic rates of change (2019–2020, 2019–2021). We used 2019 as a reference year (instead of 2020) to calculate the change to 2021, because it is the last year of pre-pandemic values to compare with a pandemic year.

We used two approaches to test whether the article production in medicine research in pandemic years (2020 & 2021) was different than in other research areas compared to previous years. First, we calculated the mean rate of change for each keyword for the four pre-pandemic rates and a confidence interval (95%), and assessed whether the pandemic rate fell within the confidence interval range: values outside the range would be considered as an abnormal increase or decrease in the number of articles published for that keyword in pandemic years, and values within the range were considered as no change in article production. We built a contingency table with the type of keywords (medicine *vs* control) and whether the pandemic rate indicated an increase, decrease, or no change. We tested for independence between type of research (medicine-related or control) and change (increase, decrease, no change), using a contingency table analysis (*i.e*., a 3 × 2 table). In our second approach, we used a linear model where the response variable was the rate of change in publications, and the fixed factors were the interaction between treatment (medicine-related and control) and era (prepandemic *vs* pandemic). The repeated linear model was run in R (R Core Team, 2016), using the lmer function in the package lme4 (Bates et al., 2015).

## Results

**Figure 1:**
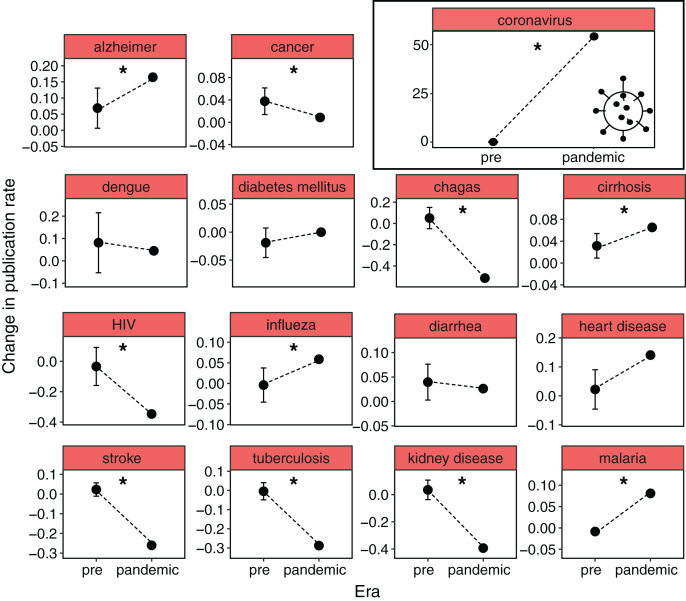
The change in publication rate in 2020 and 2021 compared to 2015–2019 decreased for most of the diseases, except for coronavirus. Pre-pandemic (pre) shows the average change in publication rate from one year to the next from 2015–2019 and the 95% CI. The pandemic value shows the mean change in the publications of 2020 and 2021 compared to 2019. An asterisk (*) denotes pandemic rate of change outside the CI range from 2015–2019.

**Figure 2:**
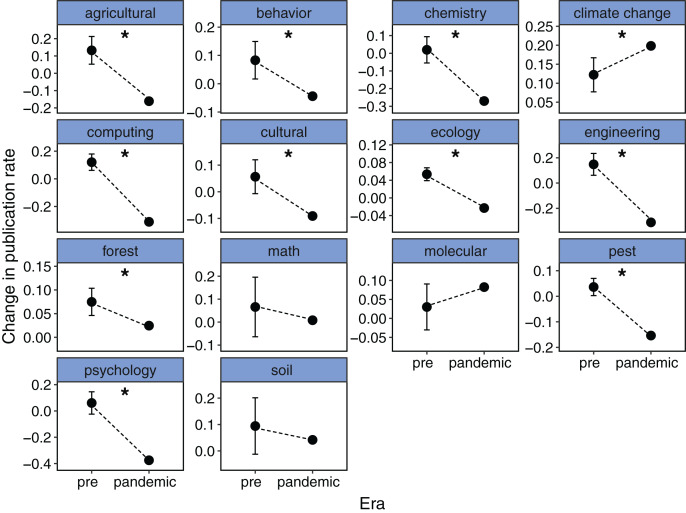
The change in publication rate in 2020 and 2021 compared to 2015–2019 decreased for most of the keywords in other non-medical research areas. Pre-pandemic (pre) shows the average change in publication rate from one year to the next from 2015–2019 and the CI. The pandemic value shows the mean change in the publications of 2020 and 2021 compared to 2019. An asterisk (*) denotes pandemic rate of change outside the CI range from 2015–2019.

**Figure 3:**
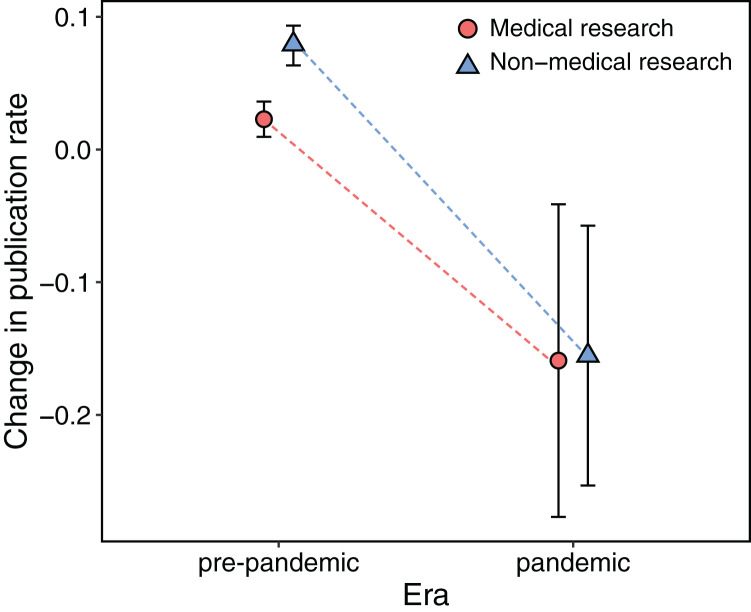
All research areas showed in average (mean ± SD) a decrease in the number of research articles in 2020 and 2021 (pandemic), compared to the annual changes from 2015 to 2019 (pre-pandemic).

The number of scientific publications about coronavirus increased about 50 times in pandemic years compared with previous years (Fig. 1). Between 2015 and 2019, the mean number of published papers about coronavirus was 900 per year (mean year-to-year publication rate of 0.03), while in 2020 and 2021, there were 40,584 and 62,804 manuscripts published, respectively. Contrary to our expectations, this huge increment of published papers on coronavirus was accompanied by a substantial decrease in the rate of scientific publications in both medical and non-medical areas. On the one hand, the change in the publication rate between pre- and pandemic years was independent of the type of research (medical or non-medical areas, *X*^2^ = 2.94, df = 2, *P* = 0.23). In medical and non-medical areas, six and 10 out of 14 keywords respectively, showed a significant decrease in the publication rate in pandemic years (Figs. 1, 2). On the other hand, the reduction in the mean rate of publications in medical and non-medical areas was similar between pre- and pandemic years (*i.e*., interaction term between pre- and pandemic years and type of research was non-significant, *F*_1,164_ = 2.46, *P* = 0.11, Fig. 3). The only factor that affected the rate of publications was the occurrence of the pandemic (*F*_1,164_ = 44.07, *P* < 0.001, Fig. 3). In medical sciences, the keywords showing a greater reduction in the publications during pandemic years (Fig. 1) were Chagas, kidney disease (49%), HIV (34%), stroke, and tuberculosis (30%). In non-medical sciences, the keywords showing the greatest reductions in pandemic years were psychology, chemistry, computing and engineering (Fig. 2).

## Discussion

Since resources are often limited and need to be distributed among multiple functions, the allocation principle proposes that assigning resources to one function may be at the expense of another (Lloyd, 1988). Accordingly, we expected that the redistribution of funds, equipment, and human resources toward COVID-19 research would negatively affect critical human diseases research more strongly than other scientific areas. However, the results did not support that hypothesis. Medical and non-medical sciences had several keywords with a reduction in the publication rate. Consequently, our results suggest that the negative effect of the pandemic was not concentrated on the medical research, but impacted other scientific areas.

Several explanations may be causing this pattern. First, the assumption that resources to respond to the COVID-19 pandemic were compromising the resources to study other diseases may be incorrect; hence, assigning resources to one function was not at the expense of another. For instance, the US National Institutes of Health received supplemental funding to assign in COVID-19 research, not necessarily compromising funds for other medical research yet (Yanow & Good, 2020), but that is not the case in other countries (Cornish, 2020). Consequently, the extraordinary restrictions and lockdowns worldwide imposed in response to the pandemic presumably caused the reduction in the publication rate regardless of subject area. Also, researchers may feel less confident to apply for non-COVID research grants (Walker et al., 2022). The restrictions imposed by the pandemic can affect all researchers in their academic and personal lives, disturbing scientific production (Korbel & Stegle, 2020). The closure of universities and research institutions, unable researchers to complete critical experiments or clinical trials required to submit scientific work for publication (Woolston, 2020b). Also, it is known that an extremely competitive environment can be detrimental for collaboration, and a shortage of funds could contribute to that environment (Anderson et al., 2007). Many researchers can lose meaningful career development opportunities with the transition of most worldwide scientific events to a virtual platform. Additionally, junior researchers may feel particularly vulnerable when sharing sensitive information and novel ideas digitally (Termini & Traver, 2020; Woolston, 2020a). Also, with the closure of universities, research institutions and schools, researchers have been compelled to work from home, in many cases overloaded with childcare, domestic responsibilities, and home-schooling (Wenham, Smith & Morgan, 2020). In sum, all these reasons probably negatively impacted the scientific activities and can explain the reduction of the publication rate that we detected in all subject areas.

The second alternative may be that the effect of restrictions imposed by the pandemic on the research activities *per se* outperforms the negative consequences of redistribution of funds, equipment, and human resources (*i.e*., the effects are not additive). In other words, the assignation of resources toward COVID-19 negatively impacted other health research programs, but this redistribution is concealed by the effect of the overall restrictions imposed by the pandemic. Finally, another explanation may be that the consequences of the allocation hypothesis are delayed and will become evident in the following years. The impact of the redistribution of resources to study COVID-19 may not be evident in a short time, as manuscripts published in 2020 and 2021 usually include experiments and data collected before the pandemic. For example, cancer research showed a slight reduction in the published papers, despite that cancer research has slowed considerably because of the fight against SARS-CoV-2 infection (Kourie et al., 2020). If this is true, research strategies, programs, and resources built over decades to prevent or eradicate other diseases are at risk of experiencing a major setback in the next future, with potentially harmful implications for our society.

Regardless of the cause, we detected a drastic reduction of published papers because of the pandemic, which hit the medical and non-medical research areas in an extraordinarily short period. The publication of scientific research is core for advancing knowledge and key for testing basic and applied ideas of how the world works. In particular, the drop in the publication rate of medical sciences delays our understanding of other diseases that strongly affect humanity, and the progress toward finding solutions. By the time we were writing this article, SARS-CoV-2 infection was responsible for almost 6.5M deaths. Nevertheless, only in 2019, heart disease, stroke, cancer, Alzheimer, diarrhea, and diabetes accounted for 23M deaths worldwide (WHO, 2020). Humans continue to be afflicted by numerous other devastating diseases besides COVID-19, and clearly, the reduction in medical research on them will be disastrous for humanity (Iacobucci, 2020). Nations should seek to catalyze the resurgence of normal research activities through specific funding opportunities to reverse the negative effect of the pandemic on the production of knowledge.

## Conclusions

Inspired in the allocation principle of ecology, we tested whether the redistribution of funds, equipment and human resources toward studying COVID-19 negatively affected the research in other diseases, compared to studies in other scientific areas. Contrary to our prediction, we found a strong decrease in the publication rate of medical and non-medical sciences during the pandemic compared to pre-pandemic years. Consequently, our results suggest that the negative effect of the pandemic was not concentrated on the medical research, but impacted other scientific areas. An injection of new resources, stronger effects of the pandemic may have prevented the allocation principle to operate. Alternatively, the stronger effects on medical sciences may take longer to become apparent.

## Supplemental Information

10.7717/peerj.15436/supp-1 Supplemental Information 1 Keywords used as search terms.

10.7717/peerj.15436/supp-2 Supplemental Information 2 Number of original research articles retrieved in the Web of Science search engine, from 2015 to 2019. In the years 2020 &2021, the number of papers each year is shown as the total ascensions for that word, and the result of excluding the papers that contained both the keyword and “covid”.

## Additional Information and Declarations

### Data Availability

The following information was supplied regarding data availability: The data is available in the Supplemental File.
