# Supplementary material for: Collateral damage: has the COVID-19 pandemic more strongly impacted medical research than other scientific areas?
Source: PeerJ. 2023 Jun 13;11:e15436. doi: 10.7717/peerj.15436 (PMC10274584; doi:10.7717/peerj.15436)
Supplement: Supplemental Information 1 [file peerj-11-15436-s001.docx]

**Table S1.** Keywords used as search terms, typed exactly as in the search engine of Web of Science.

| **Medicine-related keywords** | **Control keywords** |
| --- | --- |
| alzheimer | agricultural |
| cancer | behavior |
| chagas | chemistry |
| cirrhosis | “climate change” |
| dengue | computing |
| “diabetes mellitus” | cultural |
| diarrhea | ecology |
| “heart disease” | engineering |
| HIV | forest |
| influenza | math |
| “kidney disease” | molecular |
| malaria | pest |
| stroke | psychology |
| tuberculosis | soil |
