# Supplementary material for: Collateral damage: has the COVID-19 pandemic more strongly impacted medical research than other scientific areas?
Source: PeerJ. 2023 Jun 13;11:e15436. doi: 10.7717/peerj.15436 (PMC10274584; doi:10.7717/peerj.15436)
Supplement: Supplemental Information 2 — In the years 2020 &2021, the number of papers each year is shown as the total ascensions for that word, and the result of excluding the papers that contained both the keyword and “covid”. [file peerj-11-15436-s002.docx]

Data S1.

Number of original research articles retrieved in the Web of Science search engine, from 2015 to 2019. In the years 2020 &2021, the number of papers each year is shown as the total ascensions for that word, and the result of excluding the papers that contained both the keyword and “covid”.

| **Keyword** | **Category** | **2015** | **2016** | **2017** | **2018** | **2019** | **2020 Total** | **2020 (excluding keyword + covid)** | **2021 Total** | **2021 (excluding keyword + covid)** |
| --- | --- | --- | --- | --- | --- | --- | --- | --- | --- | --- |
| alzheimer | medical sciences | 8448 | 8733 | 9479 | 9902 | 11248 | 12386 | 12283 | 13915 | 13676 |
| cancer | medical sciences | 143812 | 152068 | 159375 | 163944 | 167700 | 164660 | 162446 | 175985 | 172083 |
| chagas | medical sciences | 1462 | 1649 | 1825 | 1828 | 1813 | 859 | 849 | 911 | 893 |
| cirrhosis | medical sciences | 5198 | 5319 | 5438 | 5696 | 5902 | 5819 | 5747 | 6755 | 6592 |
| coronavirus | medical sciences | 881 | 904 | 860 | 851 | 970 | 40584 | -- | 62804 | -- |
| dengue | medical sciences | 1774 | 2135 | 2284 | 2389 | 2417 | 2568 | 2400 | 2651 | 2346 |
| diabetes mellitus | medical sciences | 30775 | 30004 | 30027 | 29632 | 28544 | 26492 | 25207 | 31881 | 29878 |
| diarrhea | medical sciences | 4728 | 4829 | 4948 | 5163 | 5561 | 5948 | 5407 | 6008 | 5465 |
| heart disease | medical sciences | 27049 | 26583 | 28594 | 28301 | 29662 | 33395 | 32217 | 35464 | 33572 |
| HIV | medical sciences | 23313 | 24947 | 22762 | 22604 | 20576 | 13681 | 12931 | 13947 | 12511 |
| influeza | medical sciences | 6968 | 6741 | 6669 | 6870 | 6867 | 7676 | 5489 | 9052 | 5161 |
| kidney disease | medical sciences | 15136 | 16785 | 17389 | 17338 | 17495 | 9962 | 9626 | 11604 | 10875 |
| malaria | medical sciences | 4390 | 4367 | 4313 | 4299 | 4245 | 4397 | 4154 | 5024 | 4739 |
| stroke | medical sciences | 31227 | 31695 | 33434 | 33497 | 34248 | 23882 | 23166 | 27478 | 26369 |
| tuberculosis | medical sciences | 11050 | 11457 | 11318 | 11011 | 10862 | 7795 | 7510 | 7960 | 7384 |
| agricultural | non-medical sciences | 22986 | 27718 | 30508 | 34282 | 37675 | 28130 | 27969 | 35221 | 34796 |
| behavior | non-medical sciences | 198114 | 205566 | 216798 | 234016 | 261172 | 262050 | 258922 | 240355 | 232251 |
| chemistry | non-medical sciences | 201359 | 193074 | 195605 | 204458 | 218605 | 139924 | 137497 | 181569 | 176312 |
| climate change | non-medical sciences | 17166 | 19632 | 21248 | 24235 | 27194 | 29239 | 29021 | 36153 | 35507 |
| computing | non-medical sciences | 73660 | 78828 | 89845 | 103053 | 116365 | 99006 | 96426 | 63938 | 62358 |
| cultural | non-medical sciences | 14733 | 14992 | 15480 | 16657 | 18417 | 14753 | 14459 | 19022 | 18052 |
| ecology | non-medical sciences | 103052 | 109579 | 116824 | 123075 | 128447 | 113302 | 112806 | 138185 | 136920 |
| engineering | non-medical sciences | 116073 | 132558 | 162241 | 183334 | 200896 | 117975 | 117419 | 159264 | 157668 |
| forest | non-medical sciences | 29726 | 31871 | 34328 | 38176 | 40594 | 38024 | 37903 | 45282 | 44790 |
| math | non-medical sciences | 1779 | 1791 | 1777 | 1974 | 2365 | 2473 | 2469 | 2304 | 2279 |
| molecular | non-medical sciences | 339305 | 345809 | 358437 | 353990 | 384214 | 392783 | 387432 | 444288 | 431560 |
| pest | non-medical sciences | 22225 | 22507 | 23370 | 24963 | 25776 | 22013 | 21784 | 21843 | 21546 |
| psychology | non-medical sciences | 46538 | 46543 | 49408 | 55837 | 59064 | 35596 | 32217 | 41598 | 34604 |
| soil | non-medical sciences | 63931 | 67533 | 67696 | 76946 | 89594 | 84859 | 84597 | 102075 | 101165 |
